# Model of Post-traumatic Growth in Newly Traumatized vs. Retraumatized Adolescents

**DOI:** 10.3389/fpsyt.2021.682055

**Published:** 2021-09-30

**Authors:** Hannah Pazderka, Matthew RG. Brown, Caroline Beth McDonald-Harker, Andrew James Greenshaw, Vincent IO. Agyapong, Shannon Noble, Monica Mankowski, Bonnie Lee, Joy Omeje, Pamela Brett-MacLean, Deborah Terry Kitching, Leslie A. Hayduk, Peter H. Silverstone

**Affiliations:** ^1^Department of Psychiatry, University of Alberta, Edmonton, AB, Canada; ^2^Be Brave Ranch, Centre for Treatment of Child Sexual Abuse, Edmonton, AB, Canada; ^3^Department of Computing Science, University of Alberta, Edmonton, AB, Canada; ^4^Department of Sociology and Anthropology, Mount Royal University, Calgary, AB, Canada; ^5^Fort McMurray Public School District, Fort McMurray, AB, Canada; ^6^Fort McMurray Catholic School District, Fort McMurray, AB, Canada; ^7^Addictions Counselling Program, Faculty of Health Sciences, University of Lethbridge, Lethbridge, AB, Canada; ^8^Department of Sociology, University of Alberta, Edmonton, AB, Canada

**Keywords:** collective trauma, retraumatization, post-traumatic growth, adolescent, trauma informed practice, inoculation theory, stress sensitization, resilience

## Abstract

**Background:** In our analysis of adolescents affected by the 2016 Fort McMurray wildfire, we observed many negative mental health effects in individuals with a prior history of psychological trauma. Elevated rates of depression and markers of post-traumatic stress disorder (PTSD) were observed, consistent with the hypothesis that prior trauma may reduce sensitivity thresholds for later psychopathology (stress sensitization). Surprisingly, levels of anxiety did not differ based on prior trauma history, nor were retraumatized individuals at increased risk for recent (past month) suicidal ideation. These results are more suggestive of inoculation by prior trauma than stress sensitization. This led us to consider whether individuals with a prior trauma history showed evidence of Post-Traumatic Growth (PTG), a condition in which the experience of a previous trauma leads to areas of sparing or even improvement.

**Method:** To investigate this issue, we generated a structural equation model (SEM) exploring the role of anxiety in previously traumatized (*n* = 295) and wildfire trauma alone (*n* = 740) groups. Specifically, models were estimated to explore the relationship between hopelessness, anxiety, PTSD symptoms, self-efficacy and potential protective factors such as friend and family support in both groups. The model was tested using a cross-sectional sample of affected youth, comparing effects between the two groups.

**Results:** While both models produced relatively good fit, differences in the effects and chi-squared values led us to conclude that the groups are subject to different causal specifications in a number of areas, although details warrant caution pending additional investigation.

**Discussion:** We found that adolescents with a prior trauma history appear to have a more realistic appraisal of potential difficulties associated with traumatic events, and seem less reactive to potentially unsettling PTSD symptoms. They also seemed less prone to overconfidence as they got older, an effect seen in the adolescents without a history of trauma. Our findings provide preliminary evidence that the construct of anxiety may work differently in newly traumatized and retraumatized individuals, particularly in the context of mass trauma events.

## Introduction

It is generally recognized that a history of trauma in childhood and adolescence has negative effects on long-term functioning (1, 2), a finding that large, early investigations of Adverse Childhood Events (ACEs) (3) made evident. While adaptive sympathetic nervous system responses play a role in how individuals deal with short bursts of acute trauma, current thinking contends that long-term stress decreases the efficiency of reactions to stressful events due to changes in stress-related brain circuitry (4). Animal evidence suggests mediation of such effects via changes in the immune response leading to increases in cell inflammation, which subsequently influences neural circuitry (5). Supporting this hypothesis, we have recently shown long-lasting changes in subregions of the amygdala in those exposed to stress as children (6).

Consequently, youth with previous trauma may be more at risk of sympathetic overload or exhaustion. In other words, it is hypothesized that prior trauma reduces the sensitivity threshold for these children and adolescents, such that presentation of a stressor later on makes them more reactive and therefore more likely to endure negative effects. This has been coined the stress sensitization hypothesis (7), and one implication of this theory is that exposure to prior trauma will make individuals more vulnerable to mental disorders in the wake of proximal stressors (8), perhaps by complicating their ability to adjust to negative events (9). This hypothesis has been borne out in number of studies. For example, it was demonstrated that in newly recruited soldiers there is an increased risk of past 30-day major depressive episode or generalized anxiety following a stressful incident in the past year, for those individuals with a history of childhood maltreatment (8). More broadly speaking, a population-level National Epidemiological Survey (*n* = 34,653) in which it was demonstrated that there was an increased risk of mental health problems in individuals with a history of childhood adversities, with number of events correlating with increased risk (10). Similarly, the degree of exposure to the original stressor also correlates with risk, with more extreme exposure resulting in worse outcomes; for example, residents whose home was destroyed following a fireworks disaster responded more strongly to subsequent stressful life events than those whose home was spared (11). It has been suggested that adversities may need to cross a “severity threshold” to impact later stress vulnerability (10). Linking such observations to functioning of the autonomic nervous system, individuals with histories of adverse events showed higher levels of destabilized autonomic reactivity, as well as symptoms of worry, depression, and PTSD, in the wake of Covid-19 (12). These results support autonomic reactivity as the mechanism linking adversity and psychological ill-health following mass trauma.

That said, it is also possible that individuals with a history of prior trauma may in some ways be able to use their past experience to better cope with later traumatic events. This idea is central to a competing hypothesis to the stress sensitization model, known as inoculation theory (13). Inoculation theory suggests that prior experience successfully coping with stressors actually increases resistance to subsequent stress, and so ultimately has a protective effect. In an early paper discussing this effect, Bonanno (14) noted that the tendency to infer that psychological harm generally follows loss is predicated on the observation that most of the individuals that therapists see are not coping well. In fact, individuals who have encountered past trauma may have learned cognitive management strategies such that they are actually less likely to experience negative involuntary reactions, and therefore better able to navigate difficult situations – a trait sometimes referred to as post-traumatic growth (PTG). In support, Tedeschi and Calhoun (15) cite stronger, more meaningful relationships and an increased appreciation for life as potential positive consequences of past trauma. PTG appears to be strongly related to factors such as social support (16, 17), and females may show more benefits in terms of PTG than males (15). Thus, PTG appears to lie at the heart of the inoculation effect. Previous support for the inoculation hypothesis has also been demonstrated. Prior earthquake experience was shown to be associated with lower depression scores in the wake of another earthquake, although rumination was apparently unaffected (18). Similarly, another study found that flood victims who had experienced a previous flood were less likely to experience trait anxiety or weather-related distress related to personal loss in a second flood (13). A more recent study (9), also looking at flooding, compared individuals who experienced no home or property damage to those who experienced it in either a recent flood (single disaster) or both a recent and a past flood (double disaster). That study also found evidence consistent with the inoculation hypothesis, although it was less compelling (non-significant); still, it found no support for the stress sensitization hypothesis; the double disaster group was no more vulnerable to mental health issues after the flood than the single disaster group. It should be noted that while most of these inoculation studies look at previous experience with the same event, it is possible that coping strategies and reactions learned by one event can generalize to an unrelated future trauma. Norris and Murrell (13) argue that increased resistance to a new stressor may represent a type of “cross tolerance”, such that “exposure to one type of stressor prevents a different stressor from impairing performance”. In support of this contention, one study found that previous exposure to the September 11 terrorist attack moderated PTSD symptomatology in response to Shrira A et al. (19). Moreover, that study found that this effect only held for older adults who had high levels of previous trauma exposure, effectively ruling out simple maturation as a competing explanation for these findings.

### Present Study

In May 2016, the northern town of Fort McMurray, Alberta suffered a devastating wildfire which engulfed almost 600,000 hectares of land, destroyed over 2,400 buildings, and caused the evacuation of all 88,000 inhabitants.^1^ a remote northern settlement, there were only two vehicular routes out of town, and the nearest large city (Alberta's capital, Edmonton) was a 4.5 h drive away. Ironically, the only fatality of the fire was an automobile accident which occurred as a result of the mass exodus. The evacuation led to some individuals being stranded by the side of the road, although local communities stepped up with offers of water and gasoline to ensure safe passage. While the evacuees were generally greeted by hospitality from across Alberta and the rest of the country, it caused a massive amount of upheaval. As one teacher involved in the study described it, “we left for school in the morning, and didn't come home for 3 months”. For the children especially, evacuation was associated with upheaval, marked by redistribution in schools across the province which made room for new students with scarcely 2 months left in the school year. Anecdotally, at least some of the children displayed signs of trauma in the months that followed. For instance, one child was described as fearing the setting sun during the drive out of town, because they believed the wildfire was “following them”. As our statistics indicated that over 90% of the children in the groups surveyed were in or near Fort McMurray during the wildfire and forced to evacuate, it can be assumed the vast majority of our sample was affected in some way. Our study attempted to capture some of the broader psychological impacts of this event.

In a previous study on this disaster (20), which compared adolescents who had experienced a perceived psychological trauma prior to the wildfire (“prior trauma group”) with those who reported that the wildfire was their worst trauma (“wildfire group”), we found clear evidence of mental health problems associated with retraumatization. Namely, the prior trauma group showed increased rates of both depression and post-traumatic stress disorder (PTSD) symptomatology, as would be expected according to the sensitization hypothesis. However, contrary to expectations, there was no concomitant increase in rates of anxiety, and these individuals were at no increased risk for 30-day suicidal ideation. Thus, the present study explored whether, despite our previous findings of stress sensitization in those with a history of prior trauma, there were areas of sparing or PTG, reflective of some degree of inoculation.

We chose the construct of hopelessness as the dependent variable in our model comparing newly traumatized adolescents with those with a trauma history, for two reasons. First, hopelessness shows a strong and consistent association with suicide risk (21–24). Thus, to the extent that PTG is presumed a protective factor in our model [at least in terms of anxiety and suicidal ideation, as suggested by our first study (20)], a decrease in hopelessness could suggest inoculation has occurred. Second, as Chang EC (21) points out, beyond suicidal ideation, hopelessness also mediates other indices of vulnerability such as emotional dysregulation, loneliness, and problem-solving deficits. Because, in our model, hopelessness represents a lack of faith in oneself or one's ability to assert a sense of agency over one's future, it bears relevance for both anxiety and suicidal ideation.

In this study, we set out to develop a structural equation model (SEM) examining how these factors affected the likelihood of someone becoming hopeless if they had or had not experienced a prior trauma. We wanted to examine whether the two groups showed a different pattern of associations in terms of their demographic characteristics, including factors such as the support of friends or family, and markers of PTSD. Because previous research has suggested that an individual's response to trauma may, in part, depend on how manageable the stress is perceived to be (4), we also incorporated the concept of self-efficacy (feeling one could effectively tackle challenges) as a potential mediating variable. We examined potential relationships amongst the attributes by generating and testing an SEM of their presumed relationships using the data gathered during the previous study. In line with the discussion on the competing theories of sensitization and inoculation, our research question generated two competing hypotheses: Hypothesis 1, which supports the sensitization model, was that the group with a history of prior trauma would show an increase in hopelessness. Hypothesis 2, which supports the inoculation theory, was that the prior trauma group would show a decrease in this measure. Conversely, the null hypothesis predicts that there will be no differences in the magnitudes of the effects between the groups.

## Methods

### Description of Data Collection

Use of survey materials for this study was approved by the University of Alberta Health Research Ethics Boards (ethics protocol number Pro00072669). Surveys were administered to all adolescent children enrolled in junior and senior high schools in both Public and Catholic School Boards in Fort McMurray, Canada from 2017 to 2019, as part of the school system's evaluation of their post-wildfire mental health programming. Both parents and students were given the option to opt out of the study, and students could withdraw participation at any point. Data collected was online and anonymous. Information gathered from students included demographics, exposure to the wildfire, and a battery of mental health questionnaires (detailed elsewhere) (25).

Participants were excluded if they (a) fell outside of the pre-determined age range for the study (10–20 years of age); (b) if they gave inconsistent answers on the questionnaires (e.g., for positive and negative questions); and (c) if they did not answer more than 75% of the questionnaires overall. Of the 4,849 children enrolled in the 2019 school year, surveys were collected from 3,217, of which 3,041 met acceptability criteria yielding a 62.7% participation rate.

To examine the issue of retraumatization, respondents were classed into two groups the “prior trauma group” (*n* = 295) and the “wildfire group” (*n* = 740), described below, in the third year of data collection. The “prior trauma group” consisted of individuals that indicated they had experienced a more traumatizing event prior to the wildfire, while the “wildfire group” was made up of individuals that stated that the wildfire was the worst event they had sustained. This was assessed via the question: *Please select the most distressing event you have experienced* (to which the possible options were: *Fort McMurray wildfire*; *Death of someone close to you*; Injury that you suffered; *Physical assault against you*; *Sexual assault against you*.) A second question was used to determine whether that event actually happened prior to the wildfire (as several years had transpired since). This question read: “*How long has it been since the event from the previous question”*. There were eight response options for the question, ranging from the past month to more than 11 years. This method led to the elimination of over 1,789 students, because they reported that their worst trauma had occurred following the wildfire. Of the remaining students, almost one-third (28.5%) reported a previous trauma history.

As has been reported previously (20), individuals with a previous history of trauma were significantly more likely to be older, male, and – not surprisingly – been exposed to less wildfire trauma. A detailed breakdown of all of the sample characteristics of the two groups, as well as the overall sample, are presented in Table 1.

**Table 1:** Sample characteristics of the “*prior trauma group”*, the “*wildfire group”*, and the overall sample.

**Variable**	**Prior trauma group (*n* = 295)**	**Wildfire group** **(*n* = 740)**	**Total (*n* = 1,035)**
Sex (self-identified), *n* (%) Female Male Other Prefer not to say	114 (38.6) 159 (53.9) 12 (4.1) 10 (3.4)	373 (50.4) 335 (45.3) 13 (1.8) 17 (2.3)	487 (47.1) 494 (47.7) 25 (2.4) 27 (2.6)
Age (years), *n* (%) 11 12 13 14 15 16 17 18 19 Mean (SD)	4 (1.4) 43 (14.6) 49 (16.6) 48 (16.3) 59 (20.0) 40 (13.6) 47 (15.9) 4 (1.4) 1 (0.3) 14.52 (1.76)	27 (3.6) 152 (20.5) 144 (19.5) 132 (17.8) 110 (14.9) 82 (11.1) 85 (11.5) 6 (0.8) 1 (0.1) 14.04 (1.77)	31 (3.0) 195 (18.8) 193 (18.6) 180 (17.4) 169 (16.3) 122 (11.8) 132 (12.8) 10 (1.0) 2 (0.2) 14.17 (1.78)
Grade, *n* (%) 7 8 9 10 11 12 Missing Mean (SD) Junior high (gr 7–9) Senior high (gr 10–12)	44 (14.9) 46 (15.6) 52 (17.6) 58 (19.7) 46 (15.6) 48 (16.3) 1 (0.3) 9.48 (1.98) 142 (48.1) 152 (51.5)	169 (22.8) 140 (18.9) 128 (17.3) 121 (16.4) 84 (11.4) 97 (13.1) 1 (0.1) 9.11 (1.83) 437 (59.1) 302 (40.8)	213 (20.6) 186 (18.0) 180 (17.4) 179 (17.3) 130 (12.6) 145 (14.0) 2 (0.2) 9.22 (1.88) 579 (55.9) 454 (43.9)
Wildfire exposure (Y responses), *n* (%) Were you in or near Ft. McMurray during any part of the wildfire? Did you evacuate because of the fire? Was your home destroyed by the fire? Did you see the fire in person?	223 (75.6) 230 (78.0) 17 (5.8) 189 (64.1)	732 (98.9) 734 (99.2) 116 (15.7) 641 (86.6)	955 (92.3) 964 (93.1) 133 (12.9) 830 (80.2)
Total exposure (/4 items, above), *n* (%) 0 1 2 3 4 Missing	61 (20.7) 10 (3.4) 33 (11.2) 177 (60.0) 13 (4.4) 1 (0.3)	5 (0.7) 2 (0.3) 80 (10.8) 548 (74.1) 104 (14.1) 1 (0.1)	66 (6.4) 12 (1.2) 113 (10.9) 725 (70.0) 117 (11.3) 2 (0.2)
Mean (SD) Trauma history (worst trauma), *n* (%) Fort McMurray wildfire Death of someone close to you Injury that you suffered Physical assault against you Sexual assault against you Other (unidentified)	2.24 (1.26) – 107 (36.3) 22 (7.5) 6 (2.0) 36 (12.2) 124 (42.0)	3.01 (0.57) 740 (100.0) – – – – –	2.79 (0.90) 740 (71.5) 107 (10.3) 22 (2.1) 6 (0.6) 36 (3.5) 124 (12.0)

*Values in parentheses are percentages, unless otherwise indicated*.

*SD, standard deviation*.

### Measures

A detailed description of all of the questionnaires administered and their methodological characteristics is available elsewhere (25). The next section focuses only on the variables and items used for the current SEM model.

#### Wildfire Trauma Exposure

This variable consisted of four yes/no questions, which were summed to calculate a scale from 0 to 4, with higher scores being reflective of greater exposure and closer proximity to the wildfire. These questions were: *Were you in or near Fort McMurray during any part of the 2016 wildfire?; Did you evacuate because of the fire?; Was your home destroyed by the fire?; Did you see the fire in person?*

#### Friend Support/Family Support

We did not use the overall score of the Child and Youth Resilience Measure - Youth 12-question version [CYRM-12 (26)] in this paper to measure resilience; instead, we extracted a few of the items to provide more precisely focused measurements. Two items from the were used to gauge support: *My family stands by me during difficult times* (Family Support), and *My friends stand by me during difficult times* (Friend Support). Responses were coded on a 1–5 scale, with higher scores indicating greater agreement with the supportive statements. Although, arguably, additional items in the CYRM could have been used as indicators, these single indicators were chosen because other items (e.g., *I enjoy my cultural and family traditions*) are open to varying interpretations which are difficult to tease apart given the limits of our data collection, and so are less likely to demonstrate causal homogeneity, a precursor for structural equation modeling. For this reason, only these indicators were selected.

#### Anxiety

Anxiety was measured using the seven-item anxiety subscale of the Hospital Anxiety and Depression Scale [HADS (27)]. Items on the HADS are scored on a scale of 0–3. After recoding negatively keyed items, an overall score was calculated such that higher scores reflected a higher level of anxiety.

#### Self Efficacy

As we were specifically interested in the impact of anxiety on feelings of self-efficacy, we used the item *I am able to solve my problems without harming myself or others* from the CYRM-12 resilience scale, to measure self efficacy. This item was chosen not only because it reflected positive self-regard, but also because it was characteristic of an attitude of hope and optimism for the future. We considered using the entire resilience score, however closer inspection suggested that it was multifactorial. For example, some questions focus on an individual's values (e.g., *Getting an education is important to me*), some on interpersonal perceptions (e.g., *My parents know a lot about me*), some on knowledge (e.g., *I know where to go in the community to get help*), among others. In addition, we had already selected two of the items from this scale as indices of Friend Support and Family Support, which would have created specification issues.

#### PTSD

Post-traumatic stress disorder symptomatology was measured using the Child PTSD Symptom Scale [CPSS (28)]. This scale assigns a value of 1–4 for each question, based on the frequency with which each symptom is endorsed. However, while the CPSS scale generally breaks the items into three factor scores, we instead chose to represent all four categories of symptoms necessary for a diagnosis of PTSD, as defined by the DSM-V (29). Specifically, items were chosen to represent: (1) intrusive symptoms, (2) avoidance, (3) negative changes in thinking and mood, and (4) hypervigilance. Other reports have also called into question the existing three factor solution of the CPSS (30), one of which indicated a superior fit for a four factor solution (31). Choosing the best item to represent each concept allowed us to ensure that all items reflected use of effortful cognitive strategies in reducing trauma. The four items chosen were: *Having upsetting thoughts about the event that came into your head when you didn't want them to* (intrusive symptoms); *Trying not to think about, talk about, or having feelings about the event* (avoidance); *Feeling upset when you think about or hear about the event* (negative cognitions); *Being overly careful – for example, checking to see who is around you and what is around you* (hypervigilance).

#### Hopelessness

This variable was seen as central to, but distinct from, depression, in that it encompasses not only feelings of negative affect, but also a feeling of being unable to make things better going forward. One can be depressed about events that have occurred or present circumstances without necessarily feeling hopeless. Hopelessness expresses a lack of faith in oneself or one's ability to make things better in the future. The indicator selected was from the Patient Health Questionnaire [PHQ-9 (32)]: *Feeling bad about yourself - or feeling that you are a failure or that you have let yourself or your family down*.

The covariance matrix of these indicators, for each group, is available in the Supplementary Materials.

### Statistical Analysis

Structural equation modeling using maximum likelihood estimation in LISREL [linear structural relations software (33)] was employed to analyse the correlations between different concepts in our model. The primary variables of interest were Friend and Family Support, Anxiety, Self Efficacy, PTSD, and the effect of these variables on our ultimate dependent variable, level of Hopelessness.

We developed a model relating these constructs, working under the assumption that relationships would function the same way in the prior trauma group and the wildfire group. Once the model was deemed to be acceptable, we ran a stacked model in which all β and γ effects were constrained to be equal between the groups. The stacked model locates the single set of effect estimates which best matches the variables' covariance matrices for the two groups (34). In essence, this permits testing of the null hypothesis, that the magnitudes of the effects connecting the concepts are the same in both groups. Thus, inconsistencies between effects in these models represent differences in causal functioning in the two groups.

### Model Description

The current study focuses on the associations between Anxiety, Friend/Family Support, Self Efficacy, and PTSD symptoms on Hopelessness (despair in how one sees their ability to effectively manage the future). Because Anxiety did not differ between the two groups, our key question was whether it played the same role in mediating feelings of Hopelessness for those with prior trauma compared to those experiencing trauma for the first time. We were particularly interested in this relationship given the fact that the prior trauma group did not exhibit increases in recent suicidal ideation. The exogenous variables, which were posited as affecting Anxiety, were Sex (self-identified), Trauma Exposure, Friend Support, and Family Support. Age was not predicted to affect Anxiety directly, rather impacting Hopelessness via an indirect effect of altered Self Efficacy (i.e., as one ages, one feels more in control).

Our model also permits Anxiety to indirectly affect one's subjective levels of Hopelessness *via* alterations in Self Efficacy (feeling of control regarding one's future). The relationship between Anxiety and Self Efficacy was therefore modelled as a reciprocal effect, since it was predicted that Anxiety would tend to reduce Self Efficacy, while Self Efficacy could also affect Anxiety (anxious mood could undermine one's sense of self, while increased self efficacy would potentially decrease an anxious mood). The working hypothesis we adopted was that these concepts work together to determine one's level of Hopelessness. In other words, our model predicted that anxiety as a core feature of the stress response may be experienced as discomforting, but only becomes truly distressing when it begins to reflect a lack of control over one's future circumstances. Friend Support and Family Support were also predicted to impact Hopelessness via Self Efficacy, in that having others show confidence in one's ability to handle events is assumed to be key to whether one feels despair in difficult circumstances. Finally, Anxiety was also posited to affect Hopelessness indirectly via changes in PTSD. In other words, experiencing PTSD symptoms could lead the individual to feel that the future is bleak and that there is little to hope for.

In the model, measurement error variances for single indicators (described above) were held at 5%, while scale indicators (i.e., the HADS Anxiety scale, and the total score of the Trauma Exposure questions), were given 10% variance. The exogenous variables were allowed free variances and to freely covary, as were the residual variances of the individual PTSD latent variables, thereby acknowledging unmodeled sources of covariance between them.

## Results

### Model Modifications and Fit

In the original iteration of our model, all the exogenous concepts were constrained so that their direct effects only impacted Anxiety. Then, Anxiety was constrained to effect Hopelessness only through Self Efficacy and PTSD symptoms. Anxiety and Self Efficacy were allowed a reciprocal effect. Changes to the model suggested by the modification indices included the following.

The first change involved freeing the effects of Age, Friend Support, and Family Support on Self Efficacy. These effects appeared reasonable, as increased maturity and social support should both be associated with feeling confidence in problem-solving. The next change suggested by the modification indices was freeing the direct effect from Anxiety to Hopelessness. As mentioned, the original model forced effects to go from Anxiety to Hopelessness only indirectly, *via* PTSD and Self Efficacy. Freeing this effect allowed that there may be other unmodeled mechanisms besides those concepts mediating the link between Anxiety and Hopelessness, which seemed theoretically plausible. Finally, the third change involved freeing the effect from Family Support to Hopelessness and the effect from Age to Hopelessness. Freeing the connection between Family Support and Hopelessness makes logical sense because having one's family stand by them clearly impacts whether a youth believes they have let their family down. Similarly, freeing the effect from Age to Hopelessness is defensible, because as an individual approaches adulthood, more is asked of them, and they likely feel a greater weight of responsibility.

In order for a change to be implemented, it was necessary for those changes to improve model fit considerably, and to be theoretically meaningful in the context of the full causal model. Importantly, all changes suggested by the diagnostics were applied to both groups, and so were made simultaneously, though each group was permitted a separate estimate. Permitting separate estimates in each group was important because a central test of our conceptualization involved constraining the two models to have the same magnitude of effects in the corresponding model locations. These changes led to an overall adequately-fitting model for the prior trauma group (χ^2^ = 38.02, df = 29, *p* = 0.12), but a slightly poorer fit for the wildfire group (χ^2^ = 57.98, df = 29, *p* < 0.02). The other goodness-of-fit statistics did not provide evidence of concerns (root mean square error of approximation [RMSEA] = 0.036 vs. 0.037; comparative fit index [CFI] = 0.992 vs. 0.985; standardized root mean squared residual [SRMR] = 0.034 vs. 0.033 for the prior trauma and wildfire groups, respectively). The lack of clear χ^2^ fit indicated we must remain cognizant of possible misspecification in these models as well as possible mismatch in causal effects between the two groups (35). The effect estimates in these models are presented in Tables 1, 2. We consider possible differences between the groups in more detail below. Note, only results from the final model for each group are reported below.

**Table 2:** Parameter estimates of Anxiety and related variables on Hopelessness in students who said they had experienced worse trauma prior to the wildfire (“*prior trauma group*”).

			**95% Confidence interval**		
**Variable**	**Estimate**	**Std error**	**Lower**	**Upper**	***z*-value**
Sex → anxiety	−3.203	0.527	−4.23592	−2.17008	−6.079^**^
Trauma exposure → anxiety	0.347	0.217	−0.07832	0.77232	1.601
Friend support → anxiety	−0.632	0.227	−1.07692	−0.18708	−2.783^**^
Family support → anxiety	−1.050	0.297	−1.63212	−0.46788	−3.534^**^
Age → self efficacy	−0.003	0.044	−0.08924	0.08324	−0.074
**Friend Support → Self Efficacy**	0.142	0.070	0.0048	0.2792	2.018^*^
Family support → self efficacy	0.365	0.100	0.169	0.561	3.641^**^
Anxiety → self efficacy	0.017	0.049	−0.07904	0.11304	0.349
Anxiety → (PTSD1) intrusive symptoms	0.123	0.010	0.1034	0.1426	12.385^**^
Anxiety → (PTSD2) avoidance	0.131	0.012	0.10748	0.15452	10.945^**^
Anxiety → (PTSD3) negative affect	0.121	0.011	0.09944	0.14256	10.655^**^
Anxiety → (PTSD4) hypervigilance	0.152	0.012	0.12848	0.17552	12.617^**^
Age → hopelessness	0.105	0.028	0.05012	0.15988	3.706^**^
Family support → hopelessness	−0.140	0.048	−0.23408	−0.04592	−2.929^**^
Anxiety → hopelessness	0.140	0.018	0.10472	0.17528	7.781^**^
Self efficacy → hopelessness	0.004	0.041	−0.07636	0.08436	0.090
(PTSD1) Intrusive symptoms → hopelessness	−0.01	0.088	−0.18248	0.16248	−0.118
(PTSD2) Avoidance → hopelessness	−0.105	0.071	−0.24416	0.03416	−1.47
(PTSD3) Negative affect → hopelessness	0.153	0.090	−0.0234	0.3294	1.696
(PTSD4) Hypervigilance → hopelessness	0.048	0.064	−0.07744	0.17344	0.755

*Coefficients are unstandardized; bold text indicates a significant relationship that is absent in the other group*.

* *p < 0.05*.

** *p < 0.01*.

### Group Comparisons

**Figure 1:**
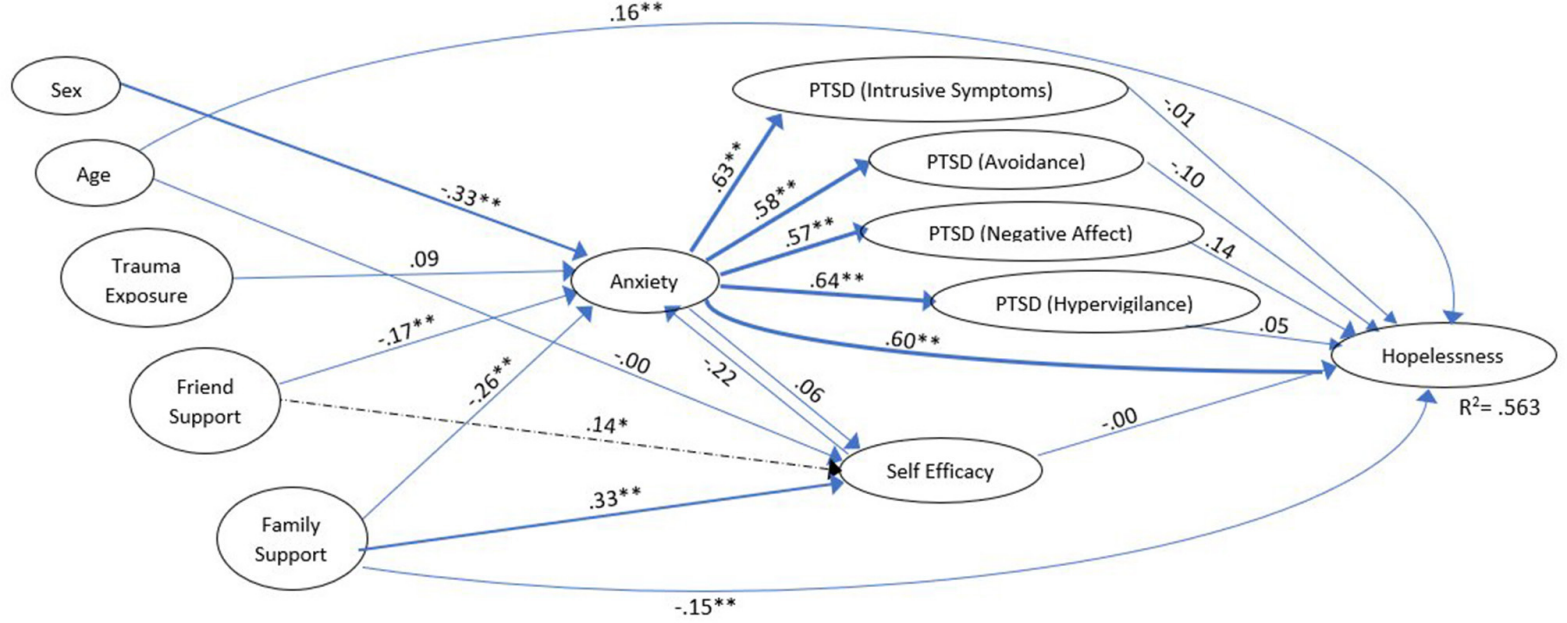
Model of adolescents who said they had experienced worse trauma prior to the wildfire (“prior trauma group”). Dashed effects are significant for the *prior trauma group only*. Coefficients are standardized for ease of comparison; effect size (36) is reflected by line weight. For clarity, the following model features have not been depicted in this figure: exogenous variable covariances, residuals on the endogenous variables, correlations among the residuals on the PTSD variables, the indicators and measurement error variances for the indicators.*p < *0.05*, **p < 0.001.

**Figure 2:**
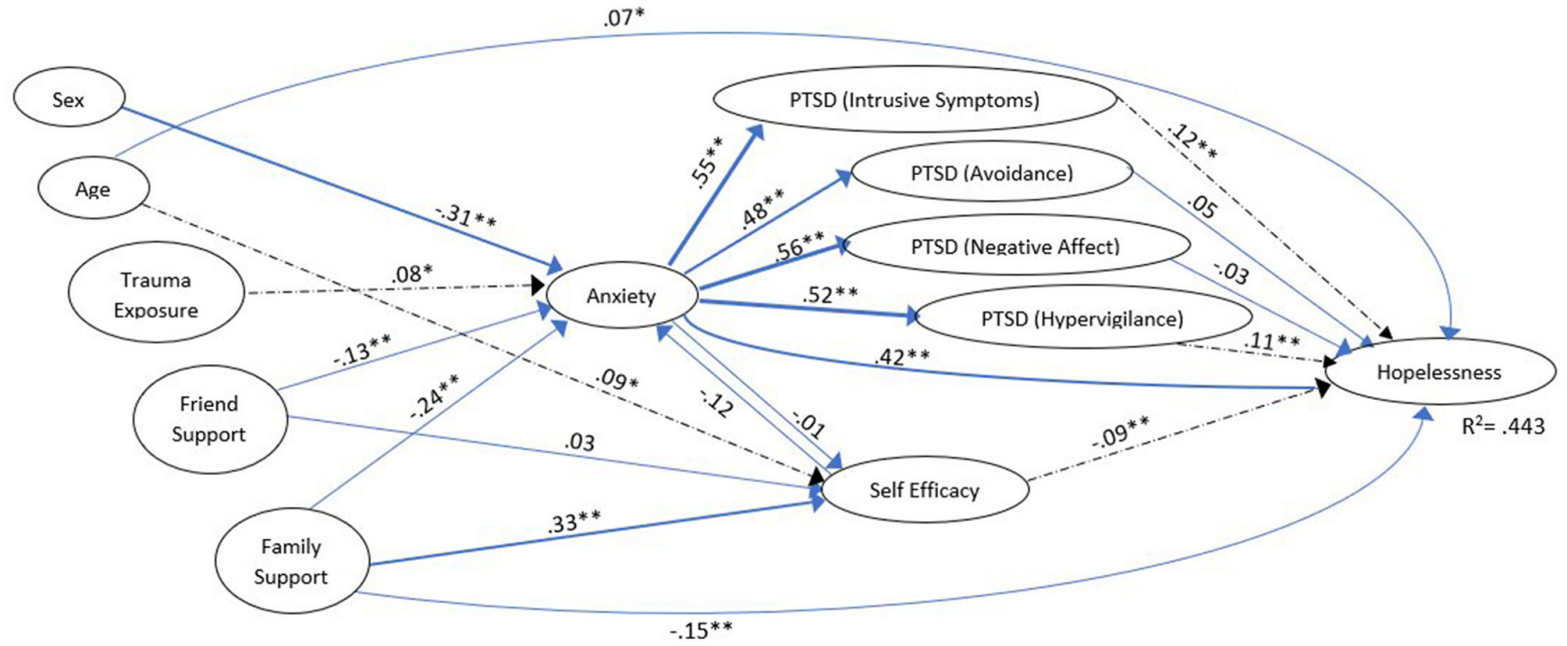
Model of adolescents who reported the 2016 wildfire as their worst trauma (“wildfire group”). Dashed effects are significant for the *wildfire group only*. Coefficients are standardized for ease of comparison; effect size (36) is reflected by line weight. For clarity, the following model features have not been depicted in this figure: exogenous variable covariances, residuals on the endogenous variables, correlations among the residuals on the PTSD variables, the indicators and measurement error variances for the indicators.*p < 0.05, **p < 0.001.

As predicted, in both groups, Friend Support and Family Support showed a significant inverse effect on Anxiety (γ = −0.166, *z* = −2.783 vs. γ = −0.125, *z* = −3.206 for Friend Support and γ = −0.260, *z* = −3.134 vs. γ = −0.241, *z* = −4.700 for Family Support (standardized effects reported throughout the text and in Figures 1, 2 which show the model for the two group, while unstandardized effects are presented in Tables 1, 2; all comparisons report prior trauma group vs. wildfire group, respectively). Similarly, stronger Family Support seemed to directly reduce Hopelessness (γ =-0148, *z* = −2.929 vs. γ = 0.148, *z* = −4.338). In other words, as level of support increases, negative consequences decrease. Sex (scored 1 = female, 2 = male) also showed a similar effect in both groups, with females reporting more Anxiety than males (γ =-0.325, *z* = −6.079 vs. γ = −0.307, *z* = −8.722).

However, there were a number of areas where the two groups differed. The effect of Trauma Exposure on Anxiety was only significant for the wildfire group (γ = 0.087, *z* = ns vs. γ = 0.078, *z* = 2.160); while the standardized effects appear quite similar, the unstandardized effects differ more clearly (Tables 2, 3). This may have been an artefact due to some of the prior trauma group having experienced relatively less exposure to the wildfire (see Table 1). However, there is no *a priori* reason to expect exposure to have a more pronounced effect on anxiety in one group compared to the other.

**Table 3:** Parameter estimates of anxiety and related variables on hopelessness in students who reported the 2016 wildfire as their worst trauma (“wildfire group”).

			**95% Confidence interval**		
**Variable**	**Estimate**	**Std error**	**Lower**	**Upper**	***z*-value**
Sex → anxiety	−2.754	0.316	−3.37336	−2.13464	−8.722^**^
**Trauma exposure → anxiety**	0.633	0.293	0.05872	1.20728	2.16^*^
Friend support → anxiety	−0.512	0.160	−0.8256	−0.1984	−3.206^**^
Family support → anxiety	−1.000	0.213	−1.41748	−0.58252	−4.700^**^
**Age → self efficacy**	0.064	0.027	0.01108	0.11692	2.393^*^
Friend support → self efficacy	0.040	0.049	−0.05604	0.13604	0.807
Family support → self efficacy	0.392	0.064	0.26656	0.51744	6.159^**^
Anxiety → self efficacy	−0.003	0.032	−0.06572	0.05972	−0.109
Anxiety → (PTSD1) intrusive symptoms	0.093	0.006	0.08124	0.10476	15.879^**^
Anxiety → (PTSD2) avoidance	0.102	0.008	0.08632	0.11768	13.394^**^
Anxiety → (PTSD3) negative affect	0.105	0.006	0.09324	0.11676	16.398^**^
Anxiety → (PTSD4) hypervigilance	0.123	0.008	0.10732	0.13868	14.827^**^
Age → hopelessness	0.039	0.017	0.00568	0.07232	2.286^*^
Family support → hopelessness	−0.138	0.032	−0.20072	−0.07528	−4.338^**^
Anxiety → hopelessness	0.093	0.010	0.0734	0.1126	8.967
**Self efficacy → hopelessness**	−0.071	0.026	−0.12196	−0.02004	−2.786^**^
**(PTSD1) Intrusive symptoms → hopelessness**	0.157	0.052	0.05508	0.25892	2.994^**^
(PTSD2) Avoidance → hopelessness	0.051	0.044	−0.03524	0.13724	1.136
(PTSD3) Negative affect → hopelessness	−0.040	0.056	−0.14976	0.06976	−0.712
**(PTSD4) Hypervigilance → hopelessness**	0.101	0.035	0.0324	0.1696	2.84^**^

*Coefficients are unstandardized; bold text indicates a significant relationship that is absent in the other group*.

* *p < 0.05*.

** *p < 0.01*.

While both groups showed a fairly strong, significant effect of Family Support on feelings of Self Efficacy (γ = 0.329, *z* = 3.641, and γ = 0.330, *z* = 6.159), only the prior trauma group showed an effect of Friend Support on this variable (γ = 0.136, *z* = 2.018 vs. γ = 0.034, *z* = ns). Thus, as Friend Support increased so did feelings of Self Efficacy. Age also had a direct effect on Self Efficacy, but only for the wildfire group (γ =-0.004, *z* = ns vs. γ = 0.088, *z* = 2.393). Thus, in the newly traumatized group older adolescents felt more confidence in solving their problems, but this did not hold true for those with severe prior trauma.

The effect of Anxiety on Self Efficacy was, unexpectedly, small and not significant for either group (γ = 0.062, *z* = ns vs. γ = −0.012, *z* = ns). Taken together, these results suggest that one's unease had less of an impact on their perceived ability to deal with problems than the support they received from others. Interestingly, the effect of Self Efficacy on Hopelessness was only significant for the wildfire group, (β = −0.004, *z* = ns vs. β = −0.091, *z* = −2.786). For this group, the more confident they felt about handling challenges, the less they reported feeling hopeless. This effect did not hold for those with prior trauma.

For both groups, Anxiety led to significant increases in all four PTSD variables, which were of approximately the same magnitude (β's > 0.571 for the prior trauma group, and β's > 0.478 for the wildfire group, all *z*'s > 10.0). Similarly, the direct effect of Anxiety on Hopelessness was significant for both groups (β = 0.598, *z* = 7.781 vs. β = 0.415, *z* = 8.976). For each of these, an increase in Anxiety meant corresponding increases in the downstream variable.

Finally, specifically examining the impact of PTSD on Hopelessness, an interesting effect emerged. For the prior trauma group, there was no effect of any of the four PTSD variables on Hopelessness (all β's <0.138, all *z*'s = ns), while for the wildfire group, two of the four PTSD dimensions – Intrusive symptoms (β = 0.120, *z* = 2.994) and Hypervigilance (β = 0.106, *z* = 2.840) – showed a significant impact on Hopelessness.

This model was found to explain 56.3% of the variance of Hopelessness in the prior trauma group, but only 44.3% in the wildfire group.

### Overall Stacked Model Comparison

Given these significant differences in the pattern of interactions between the groups, it was not surprising that the stacked model constraining the β and γ effects to be equal did not fit (χ^2^ = 127.86, df = 79, *p* < 0.001). The stacked model investigates whether some “reasonable compromise” set of effect estimates can be found that makes the data from both groups consistent with the model. Again, older criteria for other fit statistics would suggest model acceptability (RMSEA = 0.035; CFI = 0.991; SRMR = 0.039), but this does not negate the evidence of significant mismatch between the constrained model and the available data. As such, it is difficult to dismiss differences between the data and what the models predict as being due to mere random sampling variation.

We attempted freeing those β and γ effects described above as differing between the groups, but this alone did not significantly improve the chi-squared fit (χ^2^ = 123.570, df = 74, *p* < 0.001). This mismatch indicates differences in just these five effects would be insufficient to produce a fitting model. Alternately, if the two groups had been stacked with *no* constraint on the β and γ effects, then the stacked model χ^2^ would have been the sum of the individual model χ^2^'s or 96.00. That means constraining the 21 β and γ effects in the model to equality between the groups resulted in an χ^2^ increase of 31.86 with 21 degrees of freedom, which is on the borderline of statistical significance. Since we would expect many of the 21 effects to be the same in both groups, this essentially reports that more than five but somewhat fewer than all 21 modeled effects differ between the two groups. This helps verify that there is in fact a different pattern of functioning amongst individuals who have experienced prior trauma, though it is difficult to determine precisely which effects differ, and how they differ. We suggest this provides preliminary evidence that the construct of anxiety may work differently in the newly traumatized and retraumatized groups– partially due to stronger or weaker effects to or from anxiety, and partially due to somewhat different indirect effects of anxiety being transmitted though the PTSD variables as a consequence of the PTSD variables differential effects on hopelessness.

## Discussion

While our earlier research showed clear deficits in functioning in individuals who previously suffered trauma compared to the newly traumatized, a more complex analysis also suggested indications of learned skills and tendencies which may be protective. To explore this issue, we developed a structural equation model (SEM) to examine the nature of the relationship between anxiety and hopelessness in survivors of the 2,016 Fort McMurray wildfire. Fit indices suggested clear evidence of different effects between newly traumatized adolescents vs. those who had suffered an earlier trauma; however, because χ^2^ seemed to detect some lack of fit in the base model for the wildfire group, and because of some uncertainty in which specific effects are involved, we would argue these require further investigation and verification.

The relationship between Friend Support and Self Efficacy in the prior trauma group suggests that there are differences in terms of the role friend relationships play in coping for these individuals. While one might be tempted to conclude on the basis of these results that adolescents with prior trauma are more likely to seek out friends as a source of emotional support, examination of the means reveals the fact that those with a history of prior trauma are actually less likely to feel supported by friends. For example, there were over twice as many adolescents in the prior trauma group (9.6%) as compared to the wildfire group (4.1%) who responded, “*Not at all*” to the item “*My friends stand by me during difficult times*”. This may reflect the fact that adolescents with a trauma history are less trusting and so less likely to seek out support, or perhaps that they are less willing to recognize or accept support when offered. Thus, it appears the relationship between the two concepts is mainly driven by the fact that the wildfire group reported higher levels of support across the board, effectively rendering this variable a constant. That said, for those kids with prior trauma who *do* feel they have supportive friendships, there was a strengthening in how much self-efficacy this resulted in. Thus, it would appear that some retraumatized individuals derive not only comradeship, but feelings of self-efficacy from their interactions with their peers. This fits with qualitative evidence suggesting that enhanced empathy and placing greater value on relationships are important factors in promoting PTG (37), thus lowering the risk of suicidality among disaster survivors (16). It is possible that the results we obtained are, however, specific to episodes of mass trauma — it may be that, in events such as the wildfire, retraumatized adolescents were able to see others going through the same challenges (likely in contrast to their prior trauma), leading them to lean on other youth in beneficial ways. Ironically, instances of mass trauma may therefore actually provide a unique window into the development of these skills — when individuals are impacted as a group, other community members can potentially provide empathy and support in meaningful ways. This is distinctly different from individual-level trauma, in which individuals experiencing the events often feel isolated. These data suggest a role for peer support training, particularly for newly traumatized individuals. Encouragement of supportive friendships in children with a history of trauma is also strongly indicated. That said, for both newly traumatized and retraumatized youth, the relationship between Family Support, and Self Efficacy was even stronger than that observed for Friend Support, pointing to the critical role of families in helping children feel a greater sense of control when coping with trauma.

Together, relationships between Age and Self Efficacy, and Self Efficacy and Hopelessness suggest that, for individuals experiencing trauma for the first time, there exists an age-related boost in confidence in their ability to handle things, which was negatively related to Hopelessness. For the wildfire group, the oldest adolescents showed higher mean levels of confidence; for the prior trauma group, these age-related improvements did not exist. One might conclude that, for individuals with little experience with trauma, there may be a sense of overconfidence in “being able to manage” anything life throws at them, which shields these individuals from experiencing hopelessness. In one sense, this is clearly a source of strength for the newly traumatized group. However, at times, a crisis might be greater than one's capacities allow, and the overconfident adolescent might feel more distress than one who has mentally prepared for struggle. This overconfidence was not evident in the previously traumatized group, suggesting they may have developed a more realistic appraisal of trauma, given their prior experience. That said, it is worth noting that, particularly at the extremes of our age distribution, the *n*'s for these effects become quite small and represent only a few individuals; our interpretation would benefit from systematic investigation across this age range.

One possible explanation to why this overconfidence might begin to break down may be offered by the inconsistencies in how PTSD symptoms affected Hopelessness for the two groups in our model. Specifically, the two markers which differed between the groups — Hypervigilance and Intrusive Symptoms — are linked by their role in the sympathetic response. The former is associated with an increased startle reflex and exaggerated threat response, while the latter is marked by flashbacks, involuntary imagery, and dissociative reactions. Reasonably, the exhaustion that results from hypervigilance could feasibly result in an increase in intrusive thoughts. Following from this, we suggest the more this system feels out of control, the greater the feelings of perceived Hopelessness. Indeed, being on constant alert for possible dangers has the effect of leaving the individual exhausted, and ironically *more* prey to frightening, intrusive thoughts. Although rebound effects have been reported after individuals attempt to suppress negative thoughts (38) and it is also possible to interpret hypervigilance as increasing one's sense of agency via feeling as though they are “doing something” about their symptoms, our findings suggest exhaustion as a result of prolonged sympathetic activation. Importantly, this effect was observed only for the newly traumatized group in our study, and was *not* observed for those in the prior trauma group, who seem to have learned to adapt to or cope with these symptoms, lessening their ultimate impact on hopelessness. As noted by Bonanno (2004) “even among resilient individuals… virtually all participants reported intrusive cognitions and rumination at some point early after the loss” (14), which suggests the prior trauma group would have previously confronted such symptoms. In fact, individuals at risk for PTSD are often counselled to anticipate and mitigate intrusive thoughts (39).^2^ Previous experience with trauma may have taught these individuals that intrusive symptoms are to be expected — unpleasant but not necessarily threatening — and that hypervigilance is both distressing and ultimately counter-productive. That intrusive thoughts are specifically linked to sympathetic activation has been demonstrated using an analog trauma paradigm, which found that increased skin conductance (a marker of sympathetic activity) during presentation of a distressing film clip was associated with greater frequency of intrusions afterward (40). A prospective, longitudinal emergency department study in which skin conductance response (SCR) data was gathered within h of a real-life trauma event demonstrated that SCR magnitude significantly predicted which individuals would go on to develop chronic PTSD, lasting at least 1 year (41). The authors argued that their results supported the idea of sympathetic hyperactivity, perhaps leading to “overconsolidation” of distressing memories in the development of PTSD. Taken together, these findings corroborate that what we are witnessing is nervous system exhaustion in the wildfire group, with repetitive attempts (and failures) in blocking negative thoughts and feelings resulting in a sense of defeat. Thus, contrary to expectations of the sensitization model, it appears that sympathetic overactivity was most pronounced in the group newly experiencing trauma, not in retraumatized individuals. For individuals undergoing significant trauma for the first time, increased psychoeducation regarding the nature of the sympathetic response is indicated, specifically that intrusive symptoms should be anticipated, and will dissipate; that constant vigilance is not healthy; and that sympathetic overload can be effectively managed (e.g., using deep breathing, systematic relaxation, and mindfulness techniques).

For the prior trauma group, as an unfortunate consequence of resignation that intrusive thoughts are forthcoming, depression may be the unfortunate, perhaps predictable, consequence. Perhaps it is to be expected that children and adolescents who have undergone previous trauma show the expected increases in some negative variables (specifically, depression), while at the same time their previous experience has also allowed them to adapt, such that intrusive thoughts do not ultimately determine whether or not they feel like a failure. This “decoupling” could account for why these individuals do not show the expected increases in suicidal ideation that relatively often accompany depression and PTSD symptomatology. For situations of mass disaster, it may in fact be helpful to have peer support from others who have undergone significant prior trauma to discuss how some of those cognitive skills can be helpful in coping, something which the newly traumatized may not yet appreciate. In the prescient words of Norris and Murrell (13), “ ‘experienced’ victims could be a valuable resource in [mental illness] prevention efforts.”

The idea of cognitive changes being important in resilience supports the work of Huang and Gan (42), who found a relationship between PTG and positive mental associations in adolescents who had survived the Sichuan earthquake in 2008. Beyond reappraisal, researchers have also found a positive role for distraction, as supported by previous studies using negative mood induction to study the effect of either expressive or distracting creative tasks (43, 44). But how is it possible that both cognitive reappraisal and distraction can be effective in decreasing stress? It is conceivable that, as Pat-Horenczyk and Brom (45) suggest, it is “through the *oscillation between remembering and avoiding* memories of their experience [that survivors] are able to integrate the memory, find meaning in the event, and resume a balance in their functioning” (italics added). If this is the case, it may be this oscillation to which retraumatized individuals have become accustomed, which proves at least somewhat protective.

However, this interpretation should be considered in light of the fact we did not see good model fit for the wildfire group, according to χ^2^ criteria. This finding implies that, for newly traumatized adolescents, there is a need to explore the contributions of additional variables and relationships. One possibility suggested by our interpretation is that PTSD symptoms (particularly those involved in sympathetic activation) feed back to concepts of anxiety and self efficacy — an effect we posit is absent for the prior trauma group. Additionally, the incorporation of constructs such as self-esteem (self-enhancement is associated with better trauma outcomes) (14); cognitive reappraisal strategies (46); use of creative outlets, which improve positive mood (43, 44), or self-medication strategies such as alcohol or drug use, is warranted. Research is underway to examine these potential avenues.

Finally, it is worth considering why our earlier line of investigation appeared to support the stress sensitization model, while this analysis points to inoculation effects indicative of PTG. In describing inoculation theory, Norris and Murrell (13) noted that such discrepancies were evident in the literature, and suggested that prior trauma experience may be helpful in cushioning the reaction to acute traumatic events, but prove less effective in helping to manage chronic stress. However, that explanation does not seem to adequately address why we have witnessed evidence for both sensitization and inoculation in the same group of subjects in response to the same event. We speculate that it could be our use of the Hopelessness concept that has allowed us to capture elements of PTG in this data. Our conceptualization of Hopelessness focused on “lack of faith in oneself or one's ability to assert one's sense of agency”, which supports recent research finding that “hope agency” — confidence in being able to attain one's goals —accounted for additional variance in their model of suicide, over and above trauma history (47). Similarly, research has suggested that problem solving appraisal, the belief in one's capacity to effectively tackle problems, is independent of life stress in predicting suicidality (48). It is also consistent with recent research demonstrating a significant effect of both hope and trauma history, as well as their interaction, on anxiety and depression in college students (49). To this end, Clement et al. (50) note that optimism and a focus on goal-directed activity appear to be protective against suicide-related outcomes. It could be that developing a sense of hope and control over one's ability to effect positive change is an important lesson one can take away from past trauma.

In summary, differences between the prior trauma group and the wildfire group, as suggested by our model, point to some areas of PTG amongst individuals who have a prior trauma history. First, those in the prior trauma group appear more likely to benefit from a boost to self efficacy from peer support. Second, they seem less overconfident than those without a trauma history, which may be reflective of a more realistic appraisal of the challenges that accompany trauma. Third, having had firsthand experience, they may be less surprised or alarmed by symptoms of sympathetic reactivity that are associated with PTSD, such as hypervigilance or intrusive thoughts, and so may not experience the same level of anxiety as those who are newly traumatized. However, this wisdom may (quite reasonably) be accompanied by symptoms of depression. Still, the realization that one has tackled traumatic incidents before and made it through may be one of the most potent lessons that characterizes PTG in the midst of retraumatization.

## Limitations

With respect to the model itself, it should be noted that model fit is dependent on the specific indicators chosen. For example, the indicator we chose for our concept of Hopelessness primarily reflected agency, which one could argue ties it closely to conceptions of self efficacy; a different choice of indicator could relate it more closely to depression. These different indicator choices would potentially then be subject to different interpretations by the respondent. Similarly, different indicator(s) could have been chosen for each of the four PTSD variables, again with various ramifications for interpretation and response. Moreover, because relationships between these different concepts (i.e., Hopelessness and PTSD) rest on such assumptions, the overall fit of the model could theoretically differ based on these modeling decisions.

As a related issue, we noted potential issues with the use of multiple indicator scales; in particular, model specification indices suggested problems with the items comprising the concept of Anxiety. This appeared to be due to their potential overlap with some of the symptom indicators of PTSD. Such issues are commonly associated with grouping indicators based on factor analytic techniques, as has been described elsewhere (51, 52). Thus, to maximize theoretical precision, we elected to use single indicators where we determined that one item best reflected the concept of interest. That said, the reader should be aware that this method is controversial, as it can be argued that these are less reliable and more open to potential bias than validated scales. For example, if a subject misreads or misunderstands the item in question, it will produce greater modeling variability than would a scale comprised of several items. On the other hand, using a scale which is multifactorial creates a different set of problems. This is reflected in the findings described earlier by Meyer et al. (31) where a four factor solution was found to fit the PTSD data better than the three factor one suggested by the publisher. It is problematic because one may find potentially conflicting concepts driving responses to different items. So, in the questions comprising the Anxiety scale, the item *worrying thoughts go through my mind* is representative of cognitive control, while the item *I feel restless and have to be on the move* is better representative of behavioral activation. While both of these items are associated with anxiety, there is no *de facto* reason to expect them to co-occur. As we noted above, other Anxiety items (such as *I get sudden feelings of panic*) are tied fairly closely to the concept of PTSD. This overlap makes the model less clear. Hence, even this scale, which we felt was significantly robust in terms of having items that well-represented the concept, still showed evidence of modeling difficulties. Conversely, using single indicators allowed us to hone in on specific questionnaire items that we thought best captured our concepts of interest. Moreover, picking the single item best aligned with each of the four DSM-V symptom categories of PTSD allowed us to identify that a different process appeared to be at work for those symptoms mediated by sympathetic activity. This would not have been possible had we simply used the factor scores. The reader is urged to consider such implications of item selection when making modeling choices.

Individual differences are another issue which need to be considered in light of our findings. With regard to prior trauma, we did not ask individuals to rate the extent to which they felt traumatized. That is, two individuals experiencing the same event (e.g., the death of a loved one), or even experiencing the same type of event at different times or under different circumstances, can experience very different subjective events. This is important given the postulated importance of a sense of control in one's response to events (4). This would have been a useful metric to include in the model. Similarly, our findings are not meant to imply that every individual with a trauma history will show improvement, nor that it will occur in all individuals to the same extent. Indeed, it is possible that only a significant minority of patients experience PTG.

It is also a limitation that individuals were asked about prior trauma retrospectively, as recall bias may be a factor. In other words, individuals experiencing difficulty may be more likely to attribute their feelings to past negative events, while those not experiencing psychological distress may be less likely to remember such events.

Finally, while the model implications suggest some degree of inoculation and PTG in our subjects with previous trauma, our measures did not attempt to examine indicators of PTG directly. We did gather information on resilience [see Brown et al. (25) for further details], but while these two concepts are similar, they are functionally distinct. Because resilience is a considered complex and dynamic, there is no agreed-upon definition for the construct (4, 53); that said, resilience could be conceived of as an ongoing, stable dimension of human experience, with some people showing higher levels than others. Conversely, PTG occurs only in the aftermath of, and in response to, a traumatic incident, and may not happen in everyone. Future studies should consider such distinctions specifically.

## Conclusion

Between this study and our previous publication on the Fort McMurray wildfire (20), we show evidence of both sensitization and inoculation effects in the same subjects. Taken together, these papers contribute to the literature by demonstrating that both processes are likely at work at the same time. While it may be intuitive to think of individuals who have suffered previous loss and trauma primarily as victims due to their high rates of mental ill-health, this study demonstrates that, for some individuals, PTG may occur and act as a source of inoculation from the negative effects of trauma. The past experience of those with prior trauma appears to result in an improved capacity to draw personal strength from supportive peer relationships, even if those relationships are harder to come by. It may also grant these individuals the foresight to expect negative effects such as intrusive thoughts, while being able to ignore or discount the distressing hypervigilance that often attends trauma; this effectively “decouples” symptoms of sympathetic activation from emotional overreaction in those with a history of traumatic events. A trauma history may also protect individuals from overconfidence in thinking that the next distressing event will be easy to manage. While such knowledge may be inherently depressing, it is also a source of potential source of strength.

Thus, to the extent that it is hopelessness that may drive individuals to acts of desperation like self-abuse and suicide, it may be that having endured past trauma can potentially have some benefits as a protective factor, *via* the knowledge that one can withstand and overcome other difficult events in the future — a phenomenon that has been described as hope agency. This knowledge may in fact be most useful for individuals experiencing trauma for the first time, who may be particularly sensitive to the disorienting effects of sympathetic activation that occur hand-in-hand with trauma, least cognizant of the value of experienced peers, and unaware of the potential for growth that trauma provides.

## Data Availability Statement

The data analyzed in this study is subject to the following licenses/restrictions: Data for this study is the property of the Fort McMurray Public and Catholic School Boards. Requests to access these datasets should be directed to Dr. Matthew Brown, mrbrown23@gmail.com.

## Ethics Statement

The studies involving human participants were reviewed and approved by Health Research Ethics Board, University of Alberta. Written informed consent to participate in this study was provided by the participants' legal guardian/next of kin.

## Author Contributions

HP, MB, LH, and PS: study design. HP, MB, SN, MM, and DK: data collection. HP, MB, and LH: analysis. HP, MB, LH, CM-H, AG, VA, BL, JO, PB-M, and PS: manuscript preparation. All authors listed have made a substantial, direct and intellectual contribution to the work, and approved it for publication.

## Funding

Collaborative funding for this project was provided through a grant from the Canadian Institutes of Health Research, Canadian Red Cross, and Alberta Innovates Health Solutions (grant number 201600546).

## Conflict of Interest

The authors declare that the research was conducted in the absence of any commercial or financial relationships that could be construed as a potential conflict of interest.

## Publisher's Note

All claims expressed in this article are solely those of the authors and do not necessarily represent those of their affiliated organizations, or those of the publisher, the editors and the reviewers. Any product that may be evaluated in this article, or claim that may be made by its manufacturer, is not guaranteed or endorsed by the publisher.

## Footnotes

^1^ https://www.cbc.ca/news/canada/edmonton/fort-mcmurray-fire-beast-extinguished-out-1.4271604

^2^ https://www.verywellmind.com/ways-to-manage-your-ptsd-symptoms-2797613
