# Supplementary material for: Model of Post-traumatic Growth in Newly Traumatized vs. Retraumatized Adolescents
Source: Front Psychiatry. 2021 Sep 30;12:682055. doi: 10.3389/fpsyt.2021.682055 (PMC8514981; doi:10.3389/fpsyt.2021.682055)
Supplement: Supplementary file 1 [file Table_1.docx]

Table 1a. Prior trauma group: Covariance matrix

| Sex | 0.244 |  |  |  |  |  |  |  |  |  |  |  |
| --- | --- | --- | --- | --- | --- | --- | --- | --- | --- | --- | --- | --- |
| Age | 0.004 | 3.087 |  |  |  |  |  |  |  |  |  |  |
| Trauma exp. | 0.017 | 0.115 | 1.590 |  |  |  |  |  |  |  |  |  |
| Friend supp. | -0.052 | -0.007 | 0.041 | 1.642 |  |  |  |  |  |  |  |  |
| Family supp. | 0.030 | -0.176 | -0.094 | 0.485 | 1.452 |  |  |  |  |  |  |  |
| Anxiety | -0.752 | 0.962 | 0.453 | -1.630 | -2.245 | 25.032 |  |  |  |  |  |  |
| Self Efficacy | -0.005 | -0.060 | 0.013 | 0.370 | 0.533 | -1.797 | 1.784 |  |  |  |  |  |
| Hopelessness | -0.108 | 0.450 | 0.128 | -0.257 | -0.565 | 3.810 | -0.362 | 1.319 |  |  |  |  |
| Intrusions | -0.068 | 0.006 | 0.057 | -0.281 | -0.366 | 2.742 | -0.424 | 0.500 | 0.894 |  |  |  |
| Avoidance | -0.074 | 0.015 | 0.057 | -0.338 | -0.369 | 2.685 | -0.335 | 0.534 | 0.670 | 1.060 |  |  |
| Negative affect | -0.085 | -0.041 | 0.137 | -0.241 | -0.393 | 2.918 | -0.364 | 0.485 | 0.574 | 0.757 | 1.198 |  |
| Hypervigilance | -0.092 | 0.056 | 0.127 | -0.202 | -0.397 | 3.435 | -0.259 | 0.632 | 0.571 | 0.690 | 0.663 | 1.334 |

Table 1b. Wildfire group: Covariance matrix

| Sex | 0.250 |  |  |  |  |  |  |  |  |  |  |  |
| --- | --- | --- | --- | --- | --- | --- | --- | --- | --- | --- | --- | --- |
| Age | -0.081 | 3.141 |  |  |  |  |  |  |  |  |  |  |
| Trauma exp. | -0.006 | -0.003 | 0.321 |  |  |  |  |  |  |  |  |  |
| Friend supp. | -0.025 | 0.085 | -0.030 | 1.207 |  |  |  |  |  |  |  |  |
| Family supp. | 0.032 | -0.170 | -0.027 | 0.457 | 1.170 |  |  |  |  |  |  |  |
| Anxiety | -0.679 | 0.128 | 0.207 | -1.089 | -1.632 | 21.338 |  |  |  |  |  |  |
| Self Efficacy | 0.018 | 0.127 | 0.003 | 0.234 | 0.448 | -1.309 | 1.649 |  |  |  |  |  |
| Hopelessness | -0.108 | 0.105 | 0.034 | -0.191 | -0.395 | 2.638 | -0.333 | 1.013 |  |  |  |  |
| Intrusions | -0.058 | -0.158 | 0.046 | -0.131 | -0.190 | 1.782 | -0.168 | 0.321 | 0.590 |  |  |  |
| Avoidance | -0.097 | -0.122 | 0.078 | -0.104 | -0.166 | 1.997 | -0.152 | 0.306 | 0.347 | 0.708 |  |  |
| Negative affect | -0.074 | -0.066 | 0.076 | -0.114 | -0.170 | 1.956 | -0.149 | 0.330 | 0.312 | 0.503 | 0.924 |  |
| Hypervigilance | -0.067 | -0.092 | 0.082 | -0.149 | -0.190 | 2.355 | -0.141 | 0.417 | 0.323 | 0.367 | 0.424 | 1.128 |
